# Supplementary material for: IL-6, IL-12, and IL-23 STAT-Pathway Genetic Risk and Responsiveness of Lymphocytes in Patients with Multiple Sclerosis
Source: Cells. 2019 Mar 26;8(3):285. doi: 10.3390/cells8030285 (PMC6468786; doi:10.3390/cells8030285)
Supplement: Supplementary file 1 [file cells-08-00285-s001.pdf]

Supplementary Table S1

Gene	MS risk variant (SNP)	Odds ratio (OR)	Reference
IL6ST	rs7731626	1.094	[4]
IL12RB1	rs740691	1.120[1]	[4]
JAK1	rs72922276	1.164	[4]
TYK2	rs34536443	1.204	[4]
SOCS1	rs12596260	1.091[2]	[4]
STAT3	rs1026916	1.114	[4]
STAT4	rs6738544	1.074	[4]

Gene name, MS-risk variant and odds ratio used to calculate the IL-6, IL-12, and IL-23 STAT-pathway weighted genetic risk score (wGRS) of the individual donors. [1]The reference OR is for the SNP rs4808760 which is in large linkage disequilibrium (LD) with rs740691. [2]The reference OR is for the SNP rs36090551 which is in large LD with rs12596260.
